# The early development and physiology of *Xenopus laevis* tadpole lateral line system

**DOI:** 10.1152/jn.00618.2020

**Published:** 2021-10-27

**Authors:** Valentina Saccomanno, Heather Love, Amy Sylvester, Wen-Chang Li

**Affiliations:** ^1^School of Psychology and Neuroscience, University of St Andrews, Fife, United Kingdom; ^2^Department of Life Sciences, University of Trieste, Trieste, Italy

**Keywords:** afferent, efferent, hindbrain, lateral line, Xenopus laevis tadpole

## Abstract

*Xenopus laevis* has a lateral line mechanosensory system throughout its full life cycle, and a previous study on prefeeding stage tadpoles revealed that it may play a role in motor responses to both water suction and water jets. Here, we investigated the physiology of the anterior lateral line system in newly hatched tadpoles and the motor outputs induced by its activation in response to brief suction stimuli. High-speed videoing showed tadpoles tended to turn and swim away when strong suction was applied close to the head. The lateral line neuromasts were revealed by using DASPEI staining, and their inactivation with neomycin eliminated tadpole motor responses to suction. In immobilized preparations, suction or electrically stimulating the anterior lateral line nerve reliably initiated swimming but the motor nerve discharges implicating turning was observed only occasionally. The same stimulation applied during ongoing fictive swimming produced a halting response. The anterior lateral line nerve showed spontaneous afferent discharges at rest and increased activity during stimulation. Efferent activities were only recorded during tadpole fictive swimming and were largely synchronous with the ipsilateral motor nerve discharges. Finally, calcium imaging identified neurons with fluorescence increase time-locked with suction stimulation in the hindbrain and midbrain. A cluster of neurons at the entry point of the anterior lateral line nerve in the dorsolateral hindbrain had the shortest latency in their responses, supporting their potential sensory interneuron identity. Future studies need to reveal how the lateral line sensory information is processed by the central circuit to determine tadpole motor behavior.

**NEW & NOTEWORTHY** We studied *Xenopus* tadpole motor responses to anterior lateral line stimulation using high-speed videos, electrophysiology and calcium imaging. Activating the lateral line reliably started swimming. At high stimulation intensities, turning was observed behaviorally but suitable motor nerve discharges were seen only occasionally in immobilized tadpoles. Suction applied during swimming produced a halting response. We analyzed afferent and efferent activities of the tadpole anterior lateral line nerve and located sensory interneurons using calcium imaging.

## Graphical abstract

**Figure:**
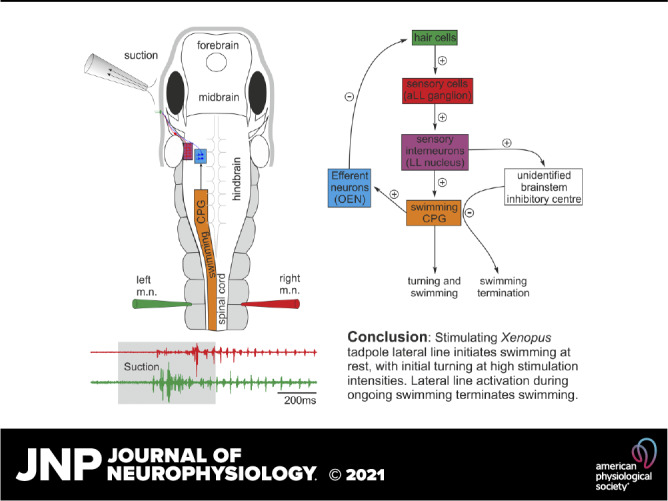

## INTRODUCTION

Aquatic vertebrates including fish and amphibians use the distributed mechanosensory lateral line (LL) system to sense hydrodynamic disturbances (1–4). The LL system provides an important sensory mode for animals to conduct rheotaxis (facing into and swimming against a current) (5–8), predation avoidance and escape (9–12), prey detection (13–15), and schooling (16–19). The LL mechanosensory neuromasts can be either located superficially in the epidermis or embedded in the lateral line canals (20). Superficial neuromasts are found in both amphibians and fish, whereas canal neuromasts are exclusive to fish (3, 21). Hair cell ciliary bundles in the neuromasts act as mechanotransducers (22). These consist of a single kinocilium and many stereovilli embedded inside a gelatinous cupula that bend with water flow (1, 23). The LL information enters the brainstem via two cranial nerves, the anterior lateral line nerve (aLLN) innervating the head region, and the posterior lateral line nerve (pLLN) innervating the trunk. Both project to a conserved nucleus in the dorsolateral medulla, the medial octavolateralis nucleus (MON) (24–30).

All anuran tadpoles possess a lateral line system before metamorphosis but only some species, like *Xenopus laevis,* retain it into adulthood (31, 32). Their LL system all have two orbital and three mandibular LL nerves, as well as trunk and tail LL nerves, with the mandibular LL nerve exhibiting more interspecific variation (33). The neuromast size, and number and organization of hair-cells, vary between species of Anura (33). Both receptor organs and sensory neurons of the LL develop from progenitor cells deposited by five LL placodes derived from the dorsolateral placode (34, 35). For *X. laevis,* LL neuromasts first appear in *stage 32* tadpoles (9, 31, 32). Unlike the adult neuromasts covered by a gelatinous cupula (36), it is thought that the cupula has not yet formed in early tadpole stages (9). Activation of the lateral line system in *X. laevis* tadpoles can initiate escape responses followed by swimming (9) and rheotactic behavior in older tadpoles (8).

With the exception of escape responses mediated by Mauthner (M) and M-homologue cells, understanding of how the central neuronal circuit processes the LL information and determines motor outputs is limited. The organization of the spinal circuit in *X. laevis* tadpoles at *stage 37*/*38* is one of the best understood and simplest among all vertebrates (37–40). Moreover, tadpole sensory modalities are limited at this stage in that only skin mechanosensory systems and the light-sensing pineal eye are functional. Other senses including ocular vision, hearing, taste, and pain are not developed (9). The simplicity of tadpole sensory and motor systems presents an excellent model for studying sensory information integration and motor decision-making at an early stage in development (41, 42). In this initial study, we investigated how the activation of the tadpole anterior LL system affected motor outputs from both a behavioral and electrophysiological perspective. We also analyzed aLLN afferent and efferent activities and located potential aLLN sensory interneurons in the brainstem with the aid of calcium imaging.

## MATERIALS AND METHODS

Mating between pairs of adult *X. laevis* was induced regularly by injections of human chorionic gonadotropin (HCG, 1,000 U/mL, Sigma, UK) into the dorsal lymph sacs. Procedures for HCG injections comply with UK Home Office regulations. All experimental procedures on tadpoles were approved by the Animal Welfare Ethics Committee (AWEC) of the University of St Andrews.

### Behavioral Tests

**Figure 1.**
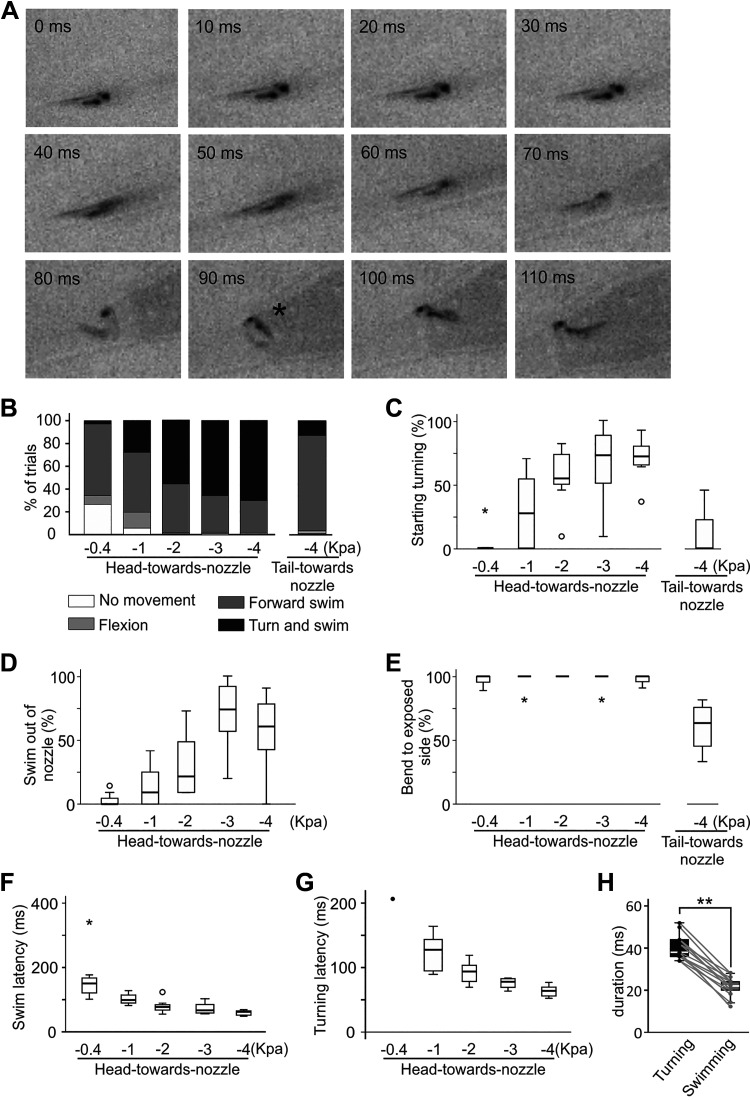
High-speed video analyses of tadpole responses to suction at different levels. *A*: single frames from a 500 fps video showing tadpole turning behavior in the head-toward-nozzle configuration at 10 ms intervals after water level starts to rise inside the suction pipette (0 ms). *The maximal bend used to estimate latencies. *B*: tadpole turning and swimming incidences increase with suction strengths in the head-toward-nozzle configuration while suction applied to tail mainly evokes swimming (tail-toward-nozzle, Pearson’s chi-squared test, *P* < 0.01, *n* = 8 tadpoles). *C*: the likelihood to evoke initial turning increases at high suction levels in the head-toward-nozzle orientation (related-samples Friedman’s two-way analysis of variance by ranks, *P* < 0.001, *n* = 8) but in the tail-toward-nozzle orientation turning incidence is lower (independent-samples Mann–Whitney *U* test for −4 kPa suction trials, *P* < 0.001, *n* = 7). *D*: the success rate for tadpoles swimming out of the suction nozzle increases with suction strength (related-samples Friedman’s two-way analysis of variance by ranks, *P* < 0.01, *n* = 8). *E*: tadpoles produce the first bend to suction consistently toward the exposed side in the head-toward-nozzle (*P* < 0.05, one-sample Wilcoxon signed rank tests to median of 100%, *n* = 7) but not in the tail-toward-nozzle orientation. *F*: the latency from the beginning of suction to the maximum swimming bend decreases with suction strength (related-samples Friedman’s two-way analysis of variance by ranks, *P* < 0.01, *n* = 8). In *C*, *E*, and *F*, circles stand for outliers and asterisks are for extremes. *G*: the latency to the maximum turning bend decreases with suction strength (related-samples Friedman’s two-way analysis of variance by ranks, *P* < 0.01, *n* = 8). *H*: turning bends have longer duration than swimming bends (paired *t* test, *n* = 13, **P < 0.001).

Skin touch activates Rohon-Beard sensory neurons innervating the skin and induces swimming in a resting tadpole (43). Before testing tadpole responses to suction, the animal tail was touched with a hair to ensure it was capable of swimming normally. A Toohey spritzer (Toohey Company, Fairfield, NJ) was used to control the timing and duration of suction, applied through a suction nozzle connected to the Toohey spritzer outlet. The inlet of the Toohey spritzer was connected to a glass bottle of 1,000 mL, the negative pressure inside which was controlled by pumping out a set amount of air using a 50-mL syringe. The suction level was calculated based on Boyle’s law (Pressure1 × Volume1 = Pressure2 × Volume2) and expressed as the difference from normal atmosphere pressure in kPa. A valve connected/disconnected the bottle with the suction nozzle, which was made of a glass pipette with an inner diameter of ∼1.5 mm. This was placed close to the tadpole lying on its side at the bottom of a 9-cm Petri dish filled with dechlorinated water. A high-speed camera (MotionBLITZ EoSens mini, Mikrotron, Germany) was used with MotionBLITZ Director2 software to capture tadpole responses to suction at 500 fps. Different suction levels were tested and for each suction setting at least 10 trials were carried out. In particular, suction stimuli of −0.4, −1, −2, −3, and −4 kPa and 500 ms long were tested in two configurations: in the “head-toward-nozzle” configuration the head was oriented toward the nozzle, whereas in the “tail-toward-nozzle” configuration the tadpole was flipped, so that in this case the tail was toward the nozzle. A turning response was defined as the tadpole contracting its myotomes and changing its body axis orientation by ∼180° outside or inside the suction pipette, whereas a flexion is characterized as a weak body bend in the middle trunk region, without propagating muscle contraction waves seen in swimming. At the beginning of each experiment, tadpoles lied at rest at the bottom of the test Petri dish in one of the aforementioned configurations. When suction started, the body axis could passively change and images could become blurry due to the tadpole entering the suction nozzle at high velocity. This made it difficult to accurately spot the first frame when tadpoles started muscle contraction. Therefore, the latency was here measured from the frame when water level started to rise inside the nozzle to the frame when the muscle contraction generated the maximal bend in the tadpole trunk (Fig. 1*A*).

### Neuromast Staining and Treatment with Neomycin

**Figure 2.**
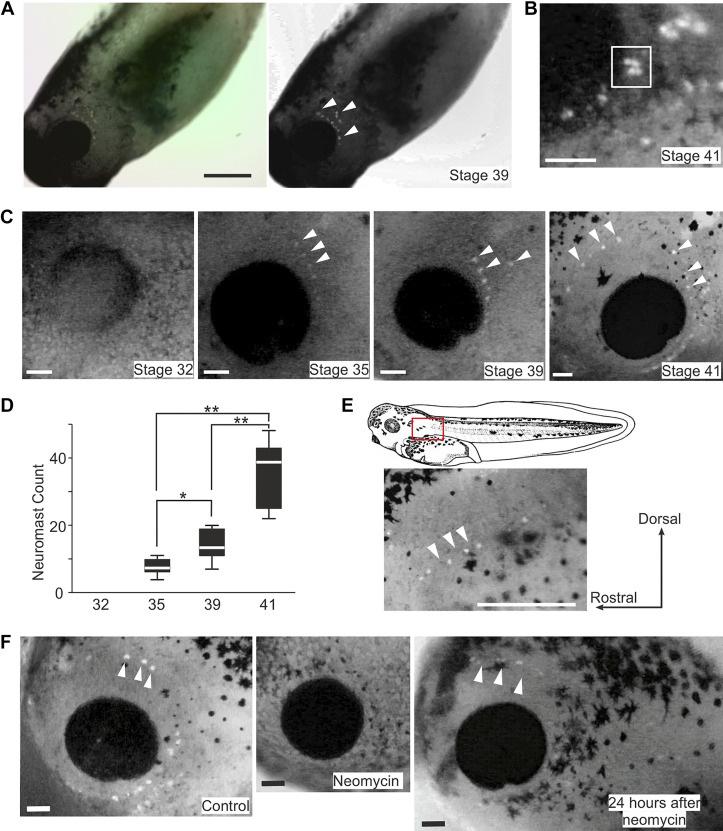
Staining tadpole lateral line neuromasts using 2-[4-(dimethylamino)styryl]-*N*-ethylpyridinium iodide (DASPEI) in control and after neomycin treatment. *A*: converting a color photo of a tadpole after DASPEI staining to gray scale after enhancing the red and yellow channels. *B*: clustering of neuromasts in a *stage 41* tadpole (square). *C*: DASPEI staining of tadpoles at various stages (only eye region shown). *D*: neuromast counts for each side of tadpoles at *stages 32*, *35*, *39*, and *41* (*n* = 10 tadpoles each, *P < 0.05 and **P < 0.01, one-way ANOVA). *E*: the appearance of posterior lateral line (LL) neuromasts. Photo shows the red rectangle area in the drawing on the *top*. *F*: DASPEI staining of *stage 40* tadpoles in control, treatment with 3 mg/mL neomycin for 30 min, and 24 h after neomycin treatment. Orientation of the tadpoles is the same in *B*, *C*, *E*, and *F*. White arrowheads point at some example neuromasts labeled with DASPEI. Scale bars: 500 µm in *A* and *E*; 100 µm in *B*, *C*, and *F*.

To visualize the LL neuromasts in tadpole skin, an assay by Pisano et al. (44) was adapted for use in the present study. Tadpoles were placed in 0.2 mg/mL solution of vital dye 2-[4-(dimethylamino)styryl]-*N*-ethylpyridinium iodide (DASPEI, Sigma-Aldrich, UK) in saline for 30 min. The treatment was carried out in darkness to prevent photobleaching and on a rocking bed to favor the uptake of the dye. The animals were then moved in a saline bath for 5 min to wash off the excess dye. In all experiments hereafter, the saline was prepared as follows: 115 mM NaCl, 3 mM KCl, 2 mM CaCl_2_, 2.4 mM NaHCO_3_, 1 mM MgCl_2_, and 10 mM HEPES, adjusted with 5 M NaOH to pH 7.4. To identify and count stained neuromasts, a stereomicroscope was modified with the addition of a barrier filter and monochromatic LED light source at 450 nm to allow fluorescent imaging. Tadpoles were placed in saline between two recessed slides. Both sides of the head and trunk were observed and photographed using an eyepiece camera and DinoCapture imaging software (Dino-Lite Europe). To enhance visualization of the neuromasts, the original fluorescent microscopy images were converted to gray scale by enhancing the red and yellow color channels (Fig. 2*A*). All images were edited using FIJI (45) or Corel PHOTO PAINT. All experiments were performed on newly hatched prefeeding *X. laevis* larvae between developmental *stages 32*–*42*, defined by Nieuwkoop and Faber (46). To inactivate the neuromast hair cells, tadpoles were placed in a 3 mg/mL solution of neomycin (Sigma-Aldrich, UK) in saline for 30 min. Tadpoles were then imaged to confirm the loss of neuromast staining. Behavioral responses to suction before and after neomycin treatment were documented without videoing.

### Extracellular Recordings

In this study, all electrophysiological experiments were carried out in tadpoles at *stages 37*–*39* (46), at a room temperature of 18°C–22°C and in saline prepared as aforementioned. Extracellular recordings of motor nerve (m.n.) activity were made using methods previously described (47). Prior to each surgical procedure, tadpoles were briefly anesthetized with 0.1% MS-222 (3-aminobenzoic acid ester, Sigma, UK) in saline for ∼20 s and secured on a rubber stage in a dissection chamber using 50 µm electrically etched tungsten pins. The pinning was performed through the notochord, approximatively at the level of the 4th and 15th post-otic myotomal segments. The dorsal fin was slit open using an etched 200-µm tungsten needle and the tadpoles were transferred to α-bungarotoxin at 10 µM for 20–30 min, where the opening of the dorsal fin skin facilitated the diffusion of the toxin to the neuromuscular junction for immobilization. Once completely immobilized, tadpoles were pinned back to the dissection chamber. Anesthetics were not used in subsequent procedures as tadpoles at this developmental stage are considered insentient.

Tadpoles at developmental *stages 37*–*39* mainly have relevant sensory and motor systems functioning to conduct simple motor behaviors like swimming, while most of the internal organs are not developed (37). The mouth and the digestive system are not formed, and a yolk sac occupies most of the ventral portion of the animal. In further dissections, the yolk belly was removed to improve later visualization of the preparation in transmitted light, while the flank skin between the 4th and 15th myotome was carefully cut with an electrically etched tungsten needle and removed with fine forceps to expose the underlying myotomes. The animal was then moved to a recording chamber and re-pinned onto a small rotatable Sylgard stage, where it was positioned and tilted so that the rostrocaudal axis was parallel to the bottom of the chamber and the dorsal side up. The bottom of the recording dish was replaced with a coverslip to allow bright-field illumination on an upright microscope. Saline in the chamber was circulated at 0.5–1 mL/min. A gap in the rubber stage allowed glass electrodes to reach both sides of the animal trunk. Glass electrodes with a tip opening of ∼60 µm filled with saline were placed on one or two muscle clefts (normally between the 5th and 6th myotomes behind the otic capsule), wherein the axons of the motoneurons lie. This allowed the recording of motor nerve activity commanding swimming. Tadpole swimming behavior is characterized by alternating waves of lateral bending that run from head to tail along the body length at frequencies between 10 and 25 Hz (48). Similarly, swimming-like pattern of m.n. bursts alternating between the left and right and propagating from head to tail can be observed in immobilized tadpoles (48). We will henceforth refer to this patterned m.n. output as fictive swimming in this paper as we did in previous publications for ease of description. A swimming cycle is the period from one m.n. burst to the subsequent one on the same side. Tadpoles are normally immotile, and their swimming episodes can be defined by the appearing and disappearing of the regular m.n. bursting at frequencies between 10 and 25 Hz. The self-sustained fictive swimming episode normally ends spontaneously after many seconds (37, 49). Synchrony, the functional significance of which remains undefined, is another rhythmic m.n. discharge pattern where the m.n. bursts from the left and right sides take place simultaneously rather than in antiphase and at twice the swimming frequency (50). In these preparations, a smaller nozzle with a diameter of ∼120 µm connected to the suction device used in behavioral tests was placed ∼150–200 µm above the left eyecup to activate the anterior lateral line system. Suction stimuli ranged from −1 to −7.5 kPa and were 0.5–1 s long, except in experiments on afferent activities, where the duration of the stimulus was extended up to 10 s. All recordings were carried out under an Olympus BX51W1 upright microscope (Olympus Microscopy, UK).

The aLLN nerve fibers run under the skin. Following immobilization in α-bungarotoxin, either the proximal or the distal end of the aLLN was isolated for recordings depending on the experimental purpose. To record the afferent component, the aLLN was severed at its entry point to the brainstem while still connected to the skin peripherally. The head skin was cut open along the midline using superfine scissors (Vannas Scissors, WPI). From this initial slit, the skin on one side of the head was delicately separated from the underlying tissues to expose the aLLN root emerging from the dorsolateral wall of the brainstem. The aLLN runs parallel and attached to the trigeminal nerve, they are just separated by a layer of connective tissue (51). The two nerves were first separated and then the aLLN root was cut using superfine scissors, while the trigeminal nerve was left intact. Recording of anterior LL nerve activity was carried out by positioning one glass electrode on the cut end with gentle suction (∼−200 Pa).

To record the aLLN efferent component, the nerve was severed distally. The aLLN is highly branched (51). Here, we mainly referred to the dorsal ramus of the nerve innervating the infraorbital region, where neuromasts are located at this stage. Once the head skin was cut in the midline and isolated from the underlying tissues as described earlier, the aLLN fibers were separated from the trigeminal nerve and the root of the trigeminal nerve was severed at the entry point to the hindbrain. The remaining aLLN was cleared from the surrounding tissues and the aforementioned nerve ramus was cut distally with superfine scissors. Once the procedure on the nerve was completed, the head skin was peeled back, with the otic capsule removed, to facilitate the positioning of the recording electrode. The same preparation was also used for the electrical stimulation of the nerve.

Electrical currents with a duration of 0.5–2 ms were applied from a DS3 Isolated Constant Current Stimulator (Digitimer, Hertfordshire, UK) to a glass suction electrode with a diameter of ∼60 µm to stimulate the aLLN. A small LED with a 15° beam angle (C503D-WAN-CCBEB151, Farnell) was used to shine white light onto the tadpole head. The pineal eye at this developmental stage is located on top of the forebrain and senses light intensity changes, before the eyes have developed. In response to such changes, pineal photoreceptors can initiate swimming (52) by activating diencephalic-mesencephalic interneurons, which project to the hindbrain motor nuclei (53). We used LED dimming to start fictive swimming in some experiments. Light dimming through the LED, electrical stimulation through DS3, and suction application through Toohey Spritzer were all controlled by TTL pulses configured in the sampling software Signal, through the Power 1401 board (CED, Cambridge, UK). A fluid flow sensor (FS1012-1001-LQ, Farnell) was added to the tubing connecting the suction nozzle. The sensor output was amplified by 10 folds by a custom-made DC amplifier and connected to the Power1401 inputs to monitor suction during experiments.

### Calcium Imaging

In immobilized tadpoles, the head skin was cut along the midline using superfine scissors to minimize the stretching and the damage to the peripheral LL organs. The hindbrain dorsal roof was opened and some ependymal cells inside the neurocoele were removed to expose neuronal somata using a tungsten dissection needle (47). Tadpoles were left further in 5 µM Fluo-4 AM (Thermo Fisher Scientific, UK) saline solution for ∼20 min in darkness. After resting the tadpole for ∼20 min, fluorescence images were captured at 10 Hz using a ×10 water immersion lens with a Neo5.5 CMOS camera and the Andor Solis software (Oxford Instruments, UK), around the time when the anterior lateral line was activated by suction. Regions of interest (ROIs) were chosen based on visible increases of fluorescence intensity following suction during video replay. A large blank area void of tissue was chosen to determine background illumination, which was subtracted from all ROI fluorescence intensity measurements. Fluorescence intensities were given as change of intensity in percentages compared with the baseline, i.e., in the absence of sensory stimulation or motor activity at the same ROIs. Any fluorescence increase lower than 5% within 1 s after suction stimulus was classified as lack of response.

### Data Analysis and Statistics

Electrophysiological recordings were first analyzed using Dataview, courtesy of Dr. William Heitler at the University of St Andrews. Initial raw data were further processed with Excel. All datasets were examined for normality first. Nonparametric statistical methods were used for those without normal distributions using SPSS 26 (IBM).

## RESULTS

### High-Speed Video Analyses of Tadpole Response to Suction

Previous study showed that tadpoles could swim away from suction in 40% of trials at developmental *stage 37* and in 60% trials at *stage 40*/*41* (9). Here, we carried out a similar test by using two different configurations and different intensities of the stimulus.

When the tadpole was placed with its head 4–6 mm away from the suction nozzle (head-toward-nozzle configuration) and suction was set at −4 kPa with a duration of 500 ms, suction only failed to evoke motor response in one trial. In the remaining cases, tadpoles either turned in situ outside the suction nozzle (before they were lifted off the bottom of the Petri dish by the suction currents), bent their body axis opposite to the direction of suction and swam away (turn and swim behavior, 27 ± 35.32%, latency: 143.1 ± 52.1 ms, Fig. 1 and Supplemental Video 1; see https://doi.org/10.6084/m9.figshare.16458843), or they were sucked inside the pipette with similar turning followed by swimming out (59 ± 30.5%, latency: 210 ± 40.8 ms, *n* = 9 tadpoles, 10–12 trials each). Forward swimming was seen in 12 ± 12.9% cases, which combined with water currents generated by the suction resulted in tadpoles getting sucked inside the nozzle and remaining inside it.

To make the suction more effective and test a wider range of suction strengths (−0.4, −1, −2, and −3 kPa), the tadpole was placed within 2 mm of the suction nozzle. With suction strengths increasing from −0.4 to −3 kPa, swimming and turning (Supplemental Video 2; see https://doi.org/10.6084/m9.figshare.16458852) became more reliable (Pearson’s χ^2^ test, *P* < 0.01, *n* = 8 tadpoles, 10–14 trials each, Fig. 1*B*). The percentage of motor responses initiating with turning increased with suction intensity (related-samples Friedman’s two-way analysis of variance by ranks, *P* < 0.001, Fig. 1*C*). At the lowest suction level, the tadpoles were sucked into the nozzle without any motor response, or produced only 1–3 weak flexions (Supplemental Video 3; see https://doi.org/10.6084/m9.figshare.16458846), or swam forward. Once inside the suction nozzle, tadpole’s forward-swimming could be stopped by clashing with the glass wall or hitting the water surface (Supplemental Video 4; see https://doi.org/10.6084/m9.figshare.16458834). The likelihood of tadpole swimming out of the suction nozzle increased with suction strengths (Supplemental Video 2, related-samples Friedman’s two-way analysis of variance by ranks, *P* < 0.01, Fig. 1*D*). There was a decrease at −4 kPa suction, which was likely due to tadpole swimming failing to overcome the strong water flow. In terms of body side where the first movement appeared (turning, swimming, or flexion), the exposed, upper side overwhelmingly generated the first bend, regardless of suction strengths (related-samples Friedman’s two-way analysis of variance by ranks, *P* = 0.64, Fig. 1*E*). The latency for forward swimming and turning decreased with suction strengths (related-samples Friedman’s two-way analysis of variance by ranks, *P* < 0.01 in both cases, Fig. 1, *F* and *G*). We measured the duration of turning and swimming bends from the frame of the initial yawing of tadpole head to the frame when the front half of tadpole trunk had straightened up. The turning bends outside or inside the suction nozzle had similar duration (40 ± 5.8 ms, *n* = 13 trials from 10 animals), which was longer than the duration of body bends during tadpole swimming (22 ± 4.9 ms, *n* = 13 trials from 10 animals, paired *t* test, *P* < 0.001, Fig. 1*H*). These results show suction stimulation could initiate forward swimming or induce occasional flexion responses at lower suction levels, whereas turning behavior becomes common with stronger stimuli and when the tadpole is in the head-toward-nozzle configuration.

We also tried to place the suction nozzle close to the tadpole tail to stimulate the lateral line hair cells sensitive to water flow in the opposite, caudo-rostral direction (tail-toward-nozzle configuration). In seven tadpoles, suction at −4 kPa evoked less initial turning responses (13 ± 20%) than trials with the same level of suction in the head-toward-nozzle configuration (70 ± 17%, independent-samples Mann–Whitney *U* test, *P* < 0.001, Fig. 1*C*), with a latency of 136 ± 62 ms. In 83 ± 21% of trials tadpoles responded with forward swimming (Supplemental Video 5; see https://doi.org/10.6084/m9.figshare.16458837), which helped the tadpole swim clear of the suction nozzle in 65 ± 32% of cases. The initial bend, either swimming or turning, was not biased toward the exposed side (60 ± 20%, *P* < 0.05, one-sample Wilcoxon signed rank tests against median of 100%, Fig. 1*E*).

After the suction was released, the water level inside the pipette normally dropped due to the gravitational pull. This outflow of water appeared to have occasionally stopped the swimming of tadpoles that had already turned around (e.g., Supplemental Video 6; see https://doi.org/10.6084/m9.figshare.16458840). Similar stopping was also observed by using a small pipette in front of a swimming tadpole to suck it up (e.g., Supplemental Video 7; see https://doi.org/10.6084/m9.figshare.16458849). This suggests that water flow in the same direction of on-going swimming may be able to stop swimming. We did not attempt to quantify this behaviorally since the water flow rate and tadpole swimming were difficult to control or track in these cases.

### DASPEI Staining and Neomycin Treatment

We next aimed to count and locate the LL neuromasts using fluorescent DASPEI staining in live tadpoles at different developmental stages. No neuromast was visible at *stage 32*. Most neuromasts became visible as lone spots at *stage 35* and *39* with some appearing to cluster together especially at *stage 41*. In the latter case, the number of separate dots in the clusters was often difficult to resolve and they were counted as one neuromast (Fig. 2*B*). The neuromast count increased progressively, rising from 7.5 ± 4 per tadpole at *stage 35* to 13.5 ± 8 at *stage 39*, and 39 ± 18 at *stage 41* (median ± IQR, *n* = 10 tadpoles for each stage, *P* < 0.001, one-way ANOVA, Fig. 2, *C* and *D*). Until *stage 39*, neuromasts were only observed on the head, mostly arranged in a single line caudal to the eye. At *stage 39*, in some cases new neuromasts began to appear posterior to the original neuromast line (Fig. 2*A*). By *stage 41*, neuromasts were observed surrounding the eye, extending to the dorsal area (Fig. 2*C*). At this stage, the posterior LL neuromasts also started to appear in a single line down the trunk, but never reaching the tail (Fig. 2*E*).

Aminoglycoside antibiotics like gentamycin and neomycin have been used in many studies to disable the lateral line hair cells (54, 55). To verify if the swimming and turning responses were initiated by the activation of tadpole LL system, neomycin was used to inactivate the neuromasts. After exposing tadpoles to 3 mg/mL neomycin for 30 min, DASPEI staining did not label any neuromasts (*n* = 5 tadpoles, Fig. 2*F*). After 24-h rest period, a mean of 28 ± 5.25 neuromasts from both left and right sides were detected using DASPEI labeling for the second time (*n* = 5 tadpoles), indicating some recovery of the LL neuromasts. The location of these neuromasts was similar to that in control tadpoles but staining did not appear as intense as before the treatment (Fig. 2*F*). Behaviorally, neomycin-exposed tadpoles lost both swimming and turning responses to suction and were passively sucked inside the nozzle. Their probability of swimming free of the nozzle at *stage 40* (suction: 4 kPa, 500 ms) decreased from 60 ± 6.3% to 0% and showed no recovery 24 h after having been transferred to normal saline (*n* = 5 tadpoles, 5 trials per tadpole in each condition). However, tadpoles responded to touch stimulation with swimming (100%, 5 trials in each condition) both immediately after neomycin treatment and after 24-h rest period. This loss of motor response to suction showed conformity with a previous study, where tadpoles with skin abrasion caudal to the eyes or treatment with lower concentrations of neomycin reduced their swimming responses to water jets (9), indicating a role of the lateral line system in mediating the observed behavior.

### Activating the Lateral Line System in Immobilized Tadpoles

**Figure 3.**
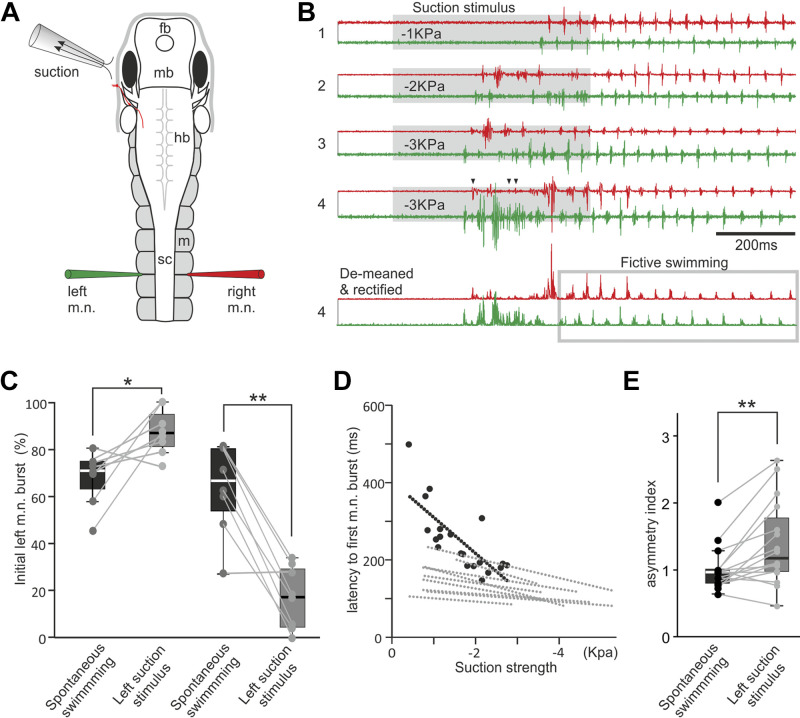
Fictive motor responses in immobilized tadpoles elicited by local suction close to the left eyecup. *A*: experimental setup diagram showing the tadpole anatomy, the position of the suction pipette, and the recording electrodes. fb, forebrain; hb, hindbrain; m, myotome; mb, midbrain; sc, spinal cord. *B*: examples of initial motor responses elicited by a 500 ms suction pulse at three different strengths (*trials 1*–*4*, gray shading) and the processing of *trial 4* recording for the calculation of the asymmetry index, which is 0.78. The gray rectangle in the demeaned and rectified *trial 4* encircles fictive swimming cycles, whose average motor nerve (m.n.) burst peak amplitude is used for normalizing the integrated m.n. activity. Traces are color-coded to match electrodes. Arrowheads indicate time with simultaneous m.n. bursts on both sides. *C*: tadpole initial m.n. bursts to suction show preference to the left (from 68 ± 11% in control to 87 ± 10%, *P* < 0.05) or right side (from 64 ± 19% in control to 17 ± 13%, *P* < 0.001, both paired *t* test, *n* = 8 in each group). *D*: latencies of first m.n. discharges decrease with suction strengths (linear regression, *P* < 0.05 in 10 tadpoles. Black circles and dotted line are for the tadpole in *B*. Gray regression lines are for 9 other tadpoles). *E*: asymmetry indices are larger in suction-evoked responses than in control (*n* = 16 tadpoles, related-samples Wilcoxon signed rank test). Significance at *P < 0.05 and **P < 0.01 in *C* and *E*.

Having located the neuromasts and established that the activation of the LL system is responsible for swimming and turning behaviors in young tadpoles, we tried to characterize these motor outputs observed behaviorally using electrophysiology in immobilized preparations. The suction nozzle used in the behavioral tests could easily move the tadpole head and activate other skin mechanosensory pathways since the tadpole was pinned down on its side to the Sylgard platform. Therefore, we chose to use a much smaller nozzle (diameter ∼120 µm) and positioned it close to the eyecup to activate neuromasts locally, while simultaneously recording the motoneuron outputs. This allowed us to determine whether we could identify any discharge pattern compatible with the results we obtained in the behavioral experiments (Fig. 3).

Behavioral tests showed that tadpoles consistently bent toward the exposed side initially, starting either swimming or turning followed by swimming. In 16 immobilized tadpoles, the initial m.n. bursts to suction stimulation showed sidedness in individual tadpoles, but not on the whole. Eight tadpoles generated bursts preferentially on the stimulated left side (Fig. 3*B*), whereas the other eight tadpoles showed increased initial bursting on the contralateral side compared with spontaneous swimming (11–25 trials each, both paired *t* test, *P* < 0.05, Fig. 3*C*). The latency of the initial m.n. responses from the start of suction also shortened with increased suction strengths in 10 of 16 tadpoles (linear regression, *P* < 0.05, Fig. 3, *B* and *D*), similar to what was observed in high-speed videos. As we showed in the high-speed videos, a turning muscle contraction lasts longer than a swimming contraction (e.g., Supplemental Video 2, Fig. 1*G*), presumably driven by prolonged discharges in the motor nerves. Although in behavioral experiments the percentage of turning increased with suction strengths, the initial m.n. activity lacked a similar progression from clear swimming rhythms to prolonged one-sided bursting implicative of turning when the suction strength was increased (*n* = 16 tadpoles). Instead, swimming was normally preceded by some prolonged bursts on one or both sides but their incidence and pattern were not correlated with suction strengths, e.g., different bursts were observed even with the same suction parameters (Fig. 3*B*, *trials 3*–*4*). In some traces, the initial m.n. response contained synchrony (50, not shown). Despite some brief, weak bursting making the initiating motor response not perfectly unilateral (arrow heads in Fig. 3*B*, *trial 4*), difference between the bursting activity on both sides would determine which side the tadpole body bends to, i.e., tadpole bends to the side with larger and more enduring m.n. bursts.

To assess and quantify the extent of such an asymmetric response, we calculated an asymmetry index (Fig. 3*B*, *trial 4*). Using Dataview software, we first subtracted the DC components from the baseline of the m.n. recording (de-meaning), rectified the traces, and integrated the m.n. activity for the period containing the prolonged bursting (Fig. 3*B*). To reduce the influence of amplitude differences between the two m.n. channels, we divided the integrated m.n. activity on each side to the average peak amplitude of the m.n. bursts produced during the first 15–20 ipsilateral fictive swimming cycles (normalization). We then calculated the asymmetry index by further dividing the normalized and integrated m.n. activity on the right side by that on the stimulated, left side. This was calculated for 5–8 responses evoked by suction at different strengths and five control swimming episodes in each tadpole. In controls, fictive swimming was either spontaneous or induced by dimming a LED light. There was no correlation between asymmetry indices and suction strengths (either Pearson correlation or Spearman’s rank correlation, *P* > 0.05 in each of 16 tadpoles examined). The average asymmetry indices were higher in case of suction (1.39 ± 0.16) than in controls (1.01 ± 0.08, *n* = 16 tadpoles, related sample Wilcoxon signed rank test, *P* < 0.01, Fig. 3*E*), indicating higher motor nerve activity on the unstimulated side before the initiation of fictive swimming.

**Figure 4.**
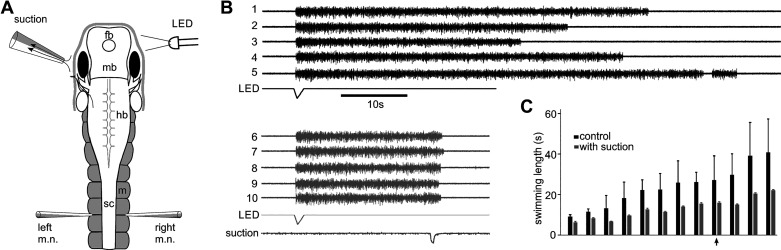
Suction stops on-going fictive swimming. *A*: experimental setup showing tadpole anatomy and the position of the LED light, suction pipette, and recording electrodes. fb, forebrain; hb, hindbrain; m, myotome; mb, midbrain; sc, spinal cord. *B*: consecutive fictive swimming episodes initiated by dimming the LED light in control (*trials 1*–*5*) and with 500 ms suction applied ∼20 s into swimming (*trials 6*–*10*). Only activity from the left motor nerve (m.n.) is shown. *C*: summary of fictive swimming lengths after suction in 12 individual tadpoles (all *P* < 0.01 except *P* = 0.012 in the tadpole indicated by an arrow, independent sample *t* test, *n* = 8–12 trials in each tadpole).

We also observed that when suction was applied in the middle of on-going fictive swimming, the fictive swimming activity was halted (Fig. 4, *A* and *B*). Normal fictive swimming episodes were 10–41 s long (*n* = 12 tadpoles). The average swimming length evoked by dimming an LED light (control) in each tadpole was estimated from 8 to 9 trials before suction was applied during on-going swimming. When we applied a 0.5-s suction stimulus at about halfway the control episode swimming length (8–12 trials in each tadpole), the episode was shortened in 127 out of 131 trials (independent sample *t* test, all *P* < 0.05, Fig. 4*C*) and only lasted 1.82 ± 0.16 s from the start of suction.

### The Effects of Electrical Stimulation of the Anterior Lateral Line Nerve

**Figure 5.**
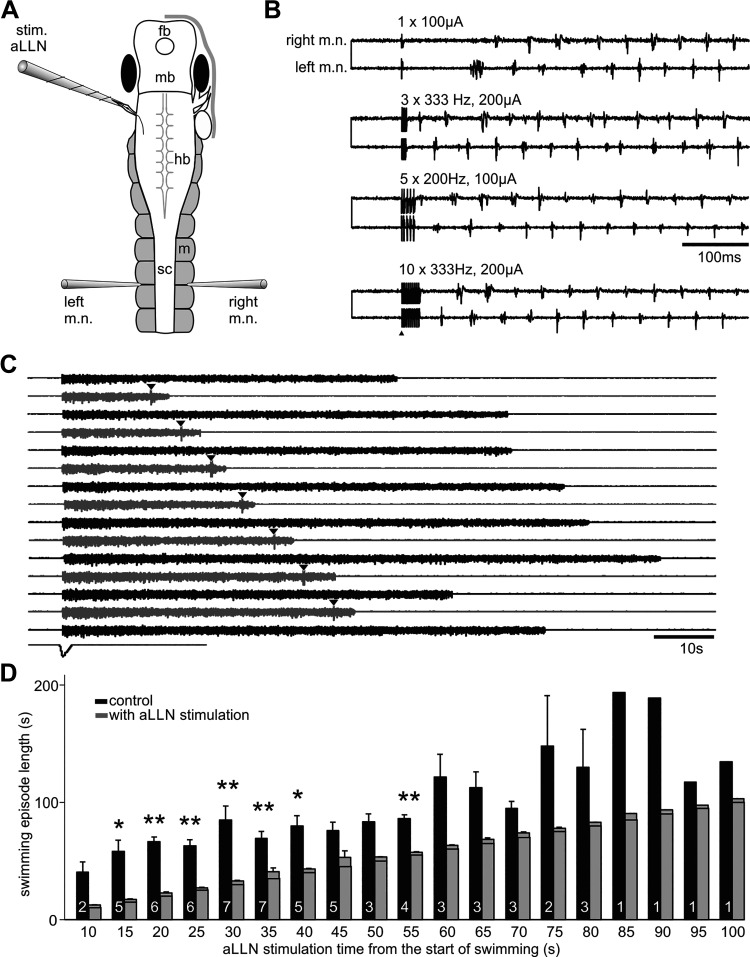
Stimulating the anterior lateral line nerve (stim. aLLN) electrically does not produce clear turning response but stops on-going fictive swimming. *A*: experimental setup showing tadpole anatomy and the position of the stimulation and recording electrodes. fb, forebrain; hb, hindbrain; m, myotome; mb, midbrain; m.n., motor nerve; sc, spinal cord. *B*: examples of stimulation of the aLLN (arrowhead; 0.5 ms pulses, other parameters given above traces) not evoking any prolonged burst before regular fictive swimming. *C*: single electrical stimulation (arrowheads) applied at various points after swimming initiation stops fictive swimming (gray traces; black traces are controls). *D*: summary of electrical stimulation of aLLN shortening fictive swimming episodes (significance at *P < 0.05, **P < 0.01, paired *t* tests). The time set for aLLN stimulation from the beginning of fictive swimming episodes in each group is also indicated by the horizontal lines inside each gray bar. Numerals inside black control bars indicate the number of tadpoles tested for each time point.

We next stimulated the ramus of the aLLN innervating the region of interest electrically to see if we could evoke similar motor responses to suction stimulation. We cut the aLLN connecting to the head skin using a pair of fine scissors and used a stimulation electrode (diameter: ∼60 µm) to suck onto the severed end. Stimulation pulses (1–20; 0.5–2 ms in duration, 20–200 µA, 200–400 Hz) were used to excite the aLLN nerve. Following stimulation, fictive swimming was initiated but without clear prolonged one-sided bursts indicating a turning response (Fig. 5). Therefore, we did not calculate the asymmetry indices. The latency of the first m.n. activity, which could be on either the stimulated or the opposite side, was 60 ± 0.02 ms (30 trials in 10 tadpoles). Then we applied aLLN stimulation at different time points of on-going fictive swimming to see if swimming could be stopped and if such stopping depended on how long swimming had carried on. The stimulation was applied during every other fictive swimming episodes so that we could carry out pair-wise comparisons of each trial with its immediate, preceding control swimming. We found that swimming could be stopped reliably at most time points tested (from 15 to 55 s into swimming, Fig. 5, *C* and *D*), just as with the more natural local suction stimuli. Fictive swimming after stimulation lasted for 3.5 ± 0.3 s, much shorter than in control conditions (with the pre-set stimulation time deducted, 47.5 ± 5.4 s, *P* < 0.001, paired *t* test, 68 trials in 9 tadpoles), but longer than the average swimming length of 1.82 s following suction (*P* < 0.01, independent-samples Median test, see section *Activating the Lateral Line System in Immobilized Tadpoles*).

### Afferent and Efferent aLLN Activity

**Figure 6.**
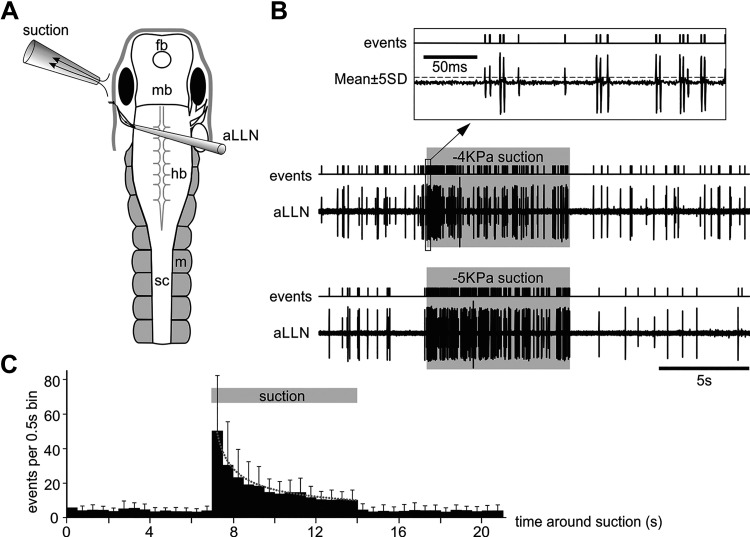
Anterior lateral line nerve (aLLN) afferent activities. *A*: experimental setup for recording aLLN afferent activity. The recording electrode directly sucks onto the cut end of aLLN. *B*: two successive examples of the aLLN activity around the time of suction (−4 kPa and −5 kPa, shaded regions). The *inset* (*top*, boxed area) shows the events triggered by setting the threshold at ±5SD in the aLLN recording trace. *C*: average binned events in 14 tadpoles showing increased activity during a 7-s suction period (bin width: 0.5 s). Dotted fitting curve is for activity during suction: *y* = 47.33 *X*^−0.61^ (*R*^2^ = 0.98).

We next focused on the afferent and efferent activities of the aLLN. To record the afferent components, the aLLN was severed at its entry point to the hindbrain. A suction pipette with ∼60 µm tip diameter sucked onto the cut end while maintaining the skin around the eye intact. A suction nozzle was placed close to the eyecup to activate the neuromasts (typical duration: 7 s, Fig. 6, *A* and *B*). In most cases, multiple units were recorded often with similar amplitudes in their extracellular action potentials. This made it difficult to clearly identify individual unitary discharges by either shapes or amplitudes, especially when the discharge frequencies were high. To simplify the analyses, we did not try to discriminate different units. Instead, we used these extracellular discharges to trigger events once they reached the threshold (set at ±5 SD of the baseline where visually identified events were reliably detected and noises excluded, Fig. 6*B*). Then we counted the number of events in each 0.5 s bin and averaged them across 14 tadpoles and 116 trials. The average number of events was higher during the suction period than before or after suction (*P* < 0.01, related-samples Wilcoxon signed rank test). The discharges to suction also decreased with time (Fig. 6*C*). This was observed at all levels of suction tested (−1 to −5 kPa). The latency from the start of suction flow to the initial first discharge was 19.7 ± 1.1 ms (13 trials in 13 tadpoles).

**Figure 7.**
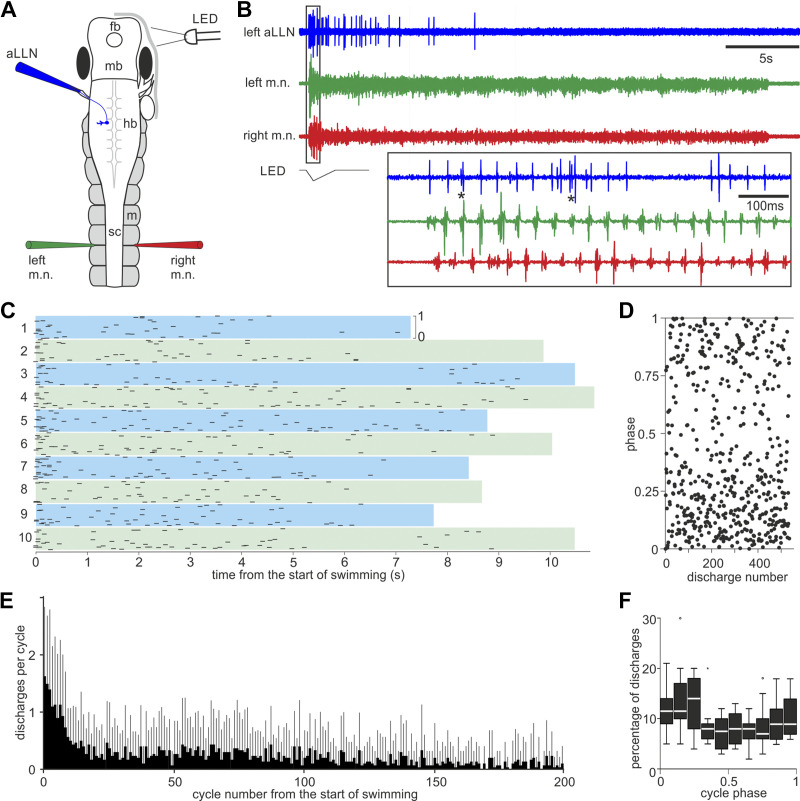
Anterior lateral line nerve (aLLN) efferent activities. *A*: setup for recording the aLLN efferent activity where fictive swimming is started by dimming an LED light. fb, forebrain; hb, hindbrain; m, myotome; mb, midbrain; sc, spinal cord. *B*: an example of aLLN efferent recording during a fictive swimming episode. The box region is expanded below to show the timing of the efferent activity relative to motor nerve (m.n.) bursts on both sides. *Examples of multiple unitary discharges within a single swimming cycle. *C*: raster plot of unitary aLLN efferent discharge phases in 10 swimming episodes from one tadpole. *Top* and *bottom* borders of color bands indicate phase from 0 to 1 as marked in *episode 1*. Color band length indicates episode duration. *D*: pooled phase plot of all 525 unitary discharges in *C*. *E*: number of unitary discharges per swimming cycle in the first 200 cycles after fictive swimming is started (averaged from 3 episodes from each of 10 tadpoles). Gray bars are SD. *F*: normalized distribution of the phases of aLLN unitary discharges calculated relative to their immediate fictive swimming cycles, defined by the m.n. bursts on the ipsilateral side at the 5th muscle cleft (100 spikes from each of 10 tadpoles, longitudinal time delays not calibrated). Peak phase is 0.11 (*Z* of Rayleigh statistic is 22.6, *P* < 0.001).

To record the aLLN efferent components, we removed the skin covering the left eyecup and cut the distal end of the nerve using a pair of fine scissors. The otic capsule was also exposed and removed, together with the trigeminal nerve. A pipette with a diameter of ∼60 µm was used to suck onto the cut end and record the aLLN efferent activity (56, 57) while the m.n. activity was recorded simultaneously (Fig. 7*A*). Efferent activity was only recorded during fictive swimming, which was initiated by dimming an LED light. As in the afferent recordings, efferent recordings also contained multiple discharges in some swimming cycles [asterix (*) in Fig. 7*B*]. We used a similar threshold-detection method to trigger discharge events and analyzed their properties. The efferent discharges were more reliable at the beginning of fictive swimming episodes and became unreliable a few seconds after fictive swimming was started, as summarized by the drop of event per swimming cycle with time (Fig. 7, *B*, *C*, and *E*). The majority of unitary discharges appeared to be rhythmic when they were reliable and were synchronous, i.e., in phase, with ipsilateral m.n. bursts during fictive swimming [Fig. 7, *B* (*inset*)–*D*]. We did not carry out rhythmicity analysis considering the possibility of discharges coming from multiple units and the lack of reliable efferent firing over long periods of time. Previously, we found that the earliest firing neurons on each swimming cycle were located in the caudal hindbrain and rostral spinal cord. There is a 3.5 ms delay over each millimeter caudally but the delay rostral to this point has not been quantified (58, 59). We did not consider this longitudinal delay when calculating the efferent activity phases. The distribution histogram of efferent unitary discharge timing showed an in-phase peak with ipsilateral (left) m.n. bursts at the 5th muscle cleft (peak phase is 0.11, *Z* of Rayleigh statistic is 22.6, *P* < 0.001, Fig. 7*F*).

### Locating Lateral Line Sensory Interneurons Using Calcium Imaging

**Figure 8.**
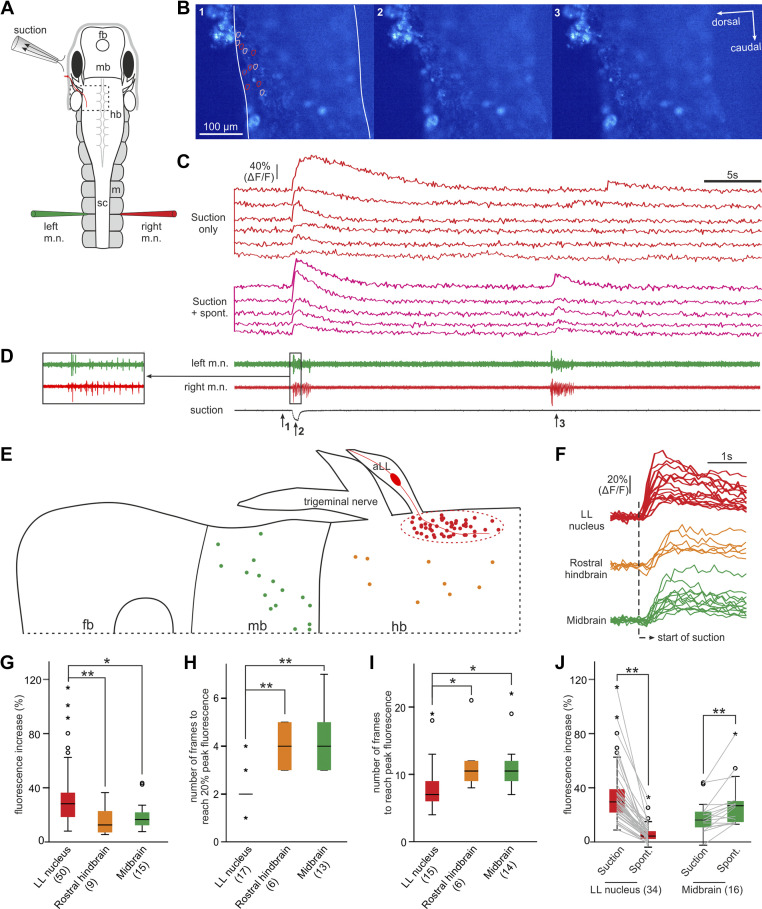
Locating anterior lateral line (aLL) nerve sensory interneurons using calcium imaging. *A*: experimental setup for calcium imaging using a ×10 water immersion objective. The brain is open and the preparation is tilted so that the left side of the hindbrain stays roughly flat to facilitate the imaging of many neurons in a single focal plane. Images have been acquired at 10 Hz. fb, forebrain; hb, hindbrain; m, myotome; mb, midbrain; sc, spinal cord. *B*: three frames captured at the indicated time points in *D* (1, 2, and 3). Lines in *frame 1* show the dorsal and ventral boundaries of the hindbrain (dotted rectangle region in *A*). Circles in *frame 1* indicate regions of interest (ROIs) where calcium activities are given in *C*. *C*: calcium activity of 11 neurons outlined by color-coded circles in *B*. Different types of responses are grouped as labeled. *D*: simultaneous motor nerve (m.n.) and suction recordings with *inset* showing initial m.n. bursts. *E*: summary of locations of neurons showing increased calcium activity time-locked with suction stimulation. *F*: examples of neurons in the lateral line (LL) nucleus region, rostral hindbrain, and midbrain to show differences in their calcium activity latencies and peak time. Summary of fluorescence peak value (*G*), number of frames to reach 20% peak fluorescence (*H*), and number of frames to reach peak fluorescence (*I*, all independent-samples Kruskal–Wallis test in *G*–*I*) in ROIs in the three brain areas following suction that has evoked swimming. *J*: comparing fluorescence increase at the beginning of swimming evoked by suction and at the beginning of spontaneous swimming (related-samples Wilcoxon signed rank test). Numbers of neurons used in statistics are given in brackets below the charts and significance levels in *G*–*J*: *P < 0.05; **P < 0.01. The same color-coding applies to *E*–*J*.

To reveal where the aLLN projects in the central nervous system, we used calcium imaging at 10 fps to locate the sensory interneurons in the brainstem on the stimulated side. We opened the dorsal roof of the 4th ventricle and removed some ependymal cells lining the inside wall of hindbrain and midbrain such that Fluo-4 AM could be loaded into the exposed neurons. After this dissection, the stub of any cut aLLN would be obscured by the hindbrain, which opened sideways when viewed from the top. This made it impractical to electrically stimulate the aLLN. Instead, we positioned a suction nozzle with a tip diameter of ∼120 µm close to the left eyecup to activate the neuromasts. A ×10 water immersion objective was used such that a large area of hindbrain and midbrain could be imaged to screen active neurons. Simultaneous m.n. and suction flow recordings were carried out (Fig. 8).

In seven *stage* *37*–*39* tadpoles, we found neurons with increased calcium activities within 1 s after the start of suction in the hindbrain and midbrain. A cluster of them was restricted in the very dorsal region where the aLLN enters the hindbrain (likely equivalent to adult LL nucleus, red and pink traces in Fig. 8*C*), whereas others were scattered more ventrally in the rostral hindbrain (Fig. 8*E*). In two of the seven tadpoles, subthreshold suction did not evoke any motor response but still led to 34.9 ± 15.9% increase of fluorescence in 24 neurons in the LL nucleus region. Suprathreshold suctions were able to initiate swimming and evoked similar levels of fluorescence increases in these neurons (32.9 ± 18.8%, related sample Wilcoxon signed rank test, *P* > 0.05).

We compared the calcium activity of the presumed LL nucleus neurons with other neurons in the hindbrain and midbrain to see if it was characteristic of sensory interneurons. Peak fluorescence in LL neurons (34.2 ± 23.8%, *n* = 50) was higher than that in rostral hindbrain neurons (16.9 ± 11.9%, *n* = 9) or in midbrain neurons (19.6 ± 11%, *n* = 15, independent-samples Kruskal–Wallis test, *P* < 0.01, Fig. 8*G*). The fluorescence normally started to rise in the first frame following suction and rose above 20% of its peak value at 2.1 ± 0.6 frames in LL neurons, in contrast to 4.0 ± 0.9 frames in rostral hindbrain and 4.3 ± 1.3 frames in midbrain neurons (independent-samples Kruskal–Wallis test, *P* < 0.01, Fig. 8, *F* and *H*). The calcium signal peaked at 8.6 ± 4.6 frames after suction in 15 LL neurons. In contrast, fluorescence peak time was at 11.8 ± 4.7 frames in rostral hindbrain neurons and at 11.5 ± 4.2 frames in midbrain neurons (independent-samples Kruskal–Wallis test, *P* < 0.05, Fig. 8*I*). In three of the seven tadpoles where spontaneous fictive swimming was also monitored, 17 out of the 34 LL neurons were also active at the start of spontaneous fictive swimming. However, the fluorescence increase in this subgroup of LL neurons was lower in the case of spontaneous swimming (11.8 ± 7.3%) compared with suction stimulation (45.2 ± 26%, Fig. 8*J*, *P* < 0.001, paired *t* test). In contrast, 17 midbrain neurons showed stronger fluorescence increase during spontaneous swimming (79.1 ± 28.3%) in comparison with suction-evoked swimming (43.5 ± 18.2%, *P* < 0.01, related-samples Wilcoxon signed rank test). Neurons in the LL nucleus therefore showed fast calcium activity following suction and weak/no activity in spontaneous swimming, supporting their identity as potential aLLN sensory interneurons.

## DISCUSSION

In this study, we first tested tadpole responses to suction behaviorally and then examined the basic physiology of aLLN fibers in immobilized tadpoles after having located the neuromasts using DASPEI labeling. The results show that the aLLN plays a role in tadpole turning behavior, initiation, and termination of swimming. In high-speed videos, initiation of swimming and turning by suction were observed reliably. Termination of on-going swimming was not examined systematically due to difficulty in implementing suction. In immobilized tadpoles, the initiation and termination of fictive swimming was reliably reproduced but motor nerve discharges implicating turning was only recorded occasionally. The discrepancies between behavioral tests and results on immobilized tadpoles likely arose from differences in experimental settings. Behavioral tests show tadpole turning away from the source of suction normally requires stronger stimulation, which might recruit more neuromasts and/or makes each neuromasts firing more vigorously. The use of smaller nozzle was necessary to avoid physically moving the tadpole in immobilized preparations, which could inadvertently activate other mechanosensory pathways. However, a smaller nozzle could only activate local neuromasts near the nozzle. This may explain the lack of clear fictive motor outputs suggestive of turning. The second method we used to activate the lateral line system was electrical stimulation of the aLLN, which also did not evoke turning-like responses. In the behavioral test, turning happens when strong suction is applied in the head-toward-nozzle configuration, while simple swimming is initiated if the suction is applied in the tail-toward-nozzle configuration. This suggests the presence of direction-selective neuromasts. Stimulating the whole nerve, indiscriminate of neuromast subtypes, may account for the reliable initiation and termination of fictive swimming and absence of fictive turning. Besides, some of the fine aLLN nerve branches may be damaged during dissection in the preparation for recordings in immobilized tadpoles.

The primary function of the tadpole LL system should lie in predator detection and escape since they are nonfeeding and inactive early in development. As similarly reported by Roberts et al. (9), our behavioral tests confirmed that tadpoles after *stage 37*/*38* can swim away from a suction stimulus. Tadpoles at *stage 37*–*40* normally lie down on their side on a bottom surface, or hang to vegetation or water surface with their cement gland. In our behavioral tests, where the tadpole lies on its side at the bottom of the Petri dish, suction will mainly stimulate the neuromasts on the exposed side before the animal moves and is sucked into the nozzle. The initial turning bend is predictably toward the exposed side, either when it happens outside or inside the nozzle. This suggests that the one-sided stimulation before tadpole starts to move with water flow may be primarily responsible for evoking the initial body bend. Turning toward the exposed side allows the tadpole to avoid head-on collision with the surface it is lying on, which could stop any motor response (47).

Turning normally ends up with tadpole changing its body axis to the opposite direction followed by swimming. In nature, this turning behavior is necessary for aquatic prey animals to swim away from the water flow generated by inertial suction-feeding predators (60). In our behavioral test, tadpoles avoided being sucked into the nozzle when they were more than 4 mm away from it. When they were closer to the nozzle, they turned inside the nozzle and managed to swim out of it in the majority of cases. In zebrafish, the LL-evoked escape is typically the C-start response, which allows larvae to avoid ∼70% of strikes (10, 11). The fast C-start in fish is mediated by Mauthner neurons, very big reticulospinal cells located at the level of the fourth rhombomere in the hindbrain (61–63). These neurons can be activated by multimodal stimuli (64), among which the LL information could be crucial for the directionality of the motor output (65). M-cells are present in *X. leavis* tadpoles by *stage 30*/*31* (66) but their functionality has never been reported so far, with the exception of their recruitment (and consequent full C-start response) at high temperature at *stage 42* (67). This is reasonable considering that many sensory modalities are underdeveloped in tadpoles at early stages to provide M-cells the proper excitation (37, 68). Nevertheless, *Xenopus* tadpoles seem to react to water currents as early as *stage 31*/*32*, coinciding with the appearance of the first neuromasts observed with a scanning electron microscope (9), and more reliably from *stage 37* to *stage 39*. This turning behavior should therefore involve other neuronal pathways rather than M-neurons. Accordingly, our recordings from immobilized tadpoles show that the initial motor response to suction has a latency of around 140 ms, or ∼60 ms after stimulating the aLLN electrically. These values are much longer than the latency for M-cell-mediated escape responses in both fish and amphibians (69, 70), even considering that tadpoles neurons at our early stage are not myelinated (71). In addition, the first motor nerve burst was recorded reliably on the stimulated side in behavioral testing and on either right or left side in immobilized tadpoles. This is not compatible with mediation by the M-cell neuronal circuit.

Apart from the role in initiating swimming and turning responses, we have revealed that activating the LL system could stop on-going fictive swimming. This opposing effect on motor output depending on whether the tadpole is active or not is similar to the response reversal observed in other preparations (72, 73). Such reversed response was also seen when the head skin was stimulated, evoking swimming at rest but terminating on-going swimming (47, 49). It will be interesting to see if the LL system and the touch sense in the head skin share the same neuronal pathways in terminating swimming.

Our experiments have revealed that the newly hatched tadpole aLLN has both afferent and efferent activities. The multiunit afferent activities show decrease with time, which could result from adaptation or some units firing transiently at the onset of suction stimulation. Although some studies revealed little or no adaptation in the lateral line afferent activity [e.g., in New Zealand longfin eel (74), toadfish (75)], adaptation has been reported in more recent studies in larval zebrafish (76, 77). Similar to what happens for other sensory systems (78, 79), adaptation might have the meaning of preventing signal saturation and retaining sensitivity to stimuli of different strength. In larval zebrafish, adaptation seems to be linked to the depletion of synaptic vesicles in hair cells leading to synaptic depression (76), similar to what happens in the auditory and vestibular systems (80, 81). Further studies are necessary to verify if the same mechanism is present in tadpoles.

The majority of LL afferents enter the caudal and medial octavolateralis nucleus in the hindbrain, called either nucleus intermedius or lateral line nucleus in amphibians (82). Some LL afferents in fish also project to the cerebellum (25, 29) and Mauthner cells (65). From the MON, the LL information is further sent to the torus semicircularis, the optic tectum, and contralateral MON via commissural connections (27, 83–87). In Anuran tadpoles, the anterior lateral line nerve projects ventromedially and the posterior lateral line nerve dorsolaterally within the ipsilateral LL nucleus (88). Using calcium imaging, we have located a number of potential sensory interneurons responding to suction stimulation with short latencies. Most of these neurons are packed within the region likely corresponding to the lateral line nucleus. While some of these neurons only showed activity in response to suction, others appeared to be also involved in the initiation of spontaneous swimming activity. When subthreshold suction was applied, neurons in this region showed activity without any motor activity. Once the stimulation was above swimming initiation threshold, their response latency was shorter than that for neurons in the rostral hindbrain and midbrain. The fluorescence increase started within the first sampling frame of 100 ms in calcium imaging after suction was applied, in line with the latency range for swimming/turning in behavioral test and initial motor nerve activity in immobilized tadpoles. The neurons in the lateral line nucleus region also demonstrated higher fluorescence increases following suction stimulus than neurons in other areas. A subgroup of them showed calcium activity in spontaneous swimming but such activity was weaker than their responses to suction. Assuming that the random loading of fluo-4 AM has not missed out any significant group of neurons responsive to suction, these data support that these neurons at the entry point of aLLN in the hindbrain are most likely the sensory interneurons. Further studies are necessary to disclose the neurochemical identity of these neurons and where they project to in the central nervous system.

In terms of LL efferents, it has been shown that both cholinergic and dopaminergic efferent neurons modulate the hair-cell output (89, 90). The cholinergic efferent system is able to reduce both spontaneous and evoked afferent activities after mechanical (57) and visual stimulation (91) or during gilling (92) and movement (93). However, in toadfish it has been recently demonstrated that efferent activities do not increase during swimming suggesting a lack of feedforward modulation of the lateral line sensory system during locomotion (75). These neurons are located in a conserved nucleus in the brainstem, the octavolateralis efferent nucleus (OEN), which projects to innervate both lateral line and inner ear hair cells in fish and amphibians (94–98). In *stages 48*–*55* tadpoles, the efferent activity was shown to be corollary to the locomotor/swimming rhythms, synchronous with the ipsilateral spinal central pattern generator activity and suppressing afferent sensory signaling (56). Consistent with this, our analyses of the efferent activity timing in *stage 37*–*39* tadpoles showed a small in-phase peak with the ipsilateral m.n. bursts. With further development, it is likely that the modulation of LL efferent activity by the ipsilateral swimming circuit will strengthen and their activities become more synchronous. There are some unidentified cholinergic neurons in the tadpole brainstem, which are involved in termination of swimming in the concussion-like events (47). We do not know if these cholinergic neurons are also candidates for giving rise to the efferents in the tadpole LL nerves, equivalent to the cholinergic OEN neurons (93, 99). Although cholinergic modulation is inhibitory, the role of dopaminergic modulation is not understood (89). In *stage 37*/*38* tadpoles, the monoaminergic systems including the dopaminergic modulation are still not endogenously functional (100). The efferent activity therefore unlikely represents dopaminergic modulation.

In summary, we have found in *Xenopus* tadpoles that the aLLN activation may play a role in their turning behavior to suction, which is followed by swimming. However, suction applied during on-going fictive swimming terminates the swimming activity. Future studies need to trace where the lateral line sensory interneurons project to in the central nervous system and how the LL sensory information is further processed to lead to these motor outputs.

## SUPPLEMENTAL DATA

10.6084/m9.figshare.16458843 Supplemental Video 1: https://doi.org/10.6084/m9.figshare.16458843.

10.6084/m9.figshare.16458852 Supplemental Video 2: https://doi.org/10.6084/m9.figshare.16458852.

10.6084/m9.figshare.16458846 Supplemental Video 3: https://doi.org/10.6084/m9.figshare.16458846.

10.6084/m9.figshare.16458834 Supplemental Video 4: https://doi.org/10.6084/m9.figshare.16458834.

10.6084/m9.figshare.16458837 Supplemental Video 5: https://doi.org/10.6084/m9.figshare.16458837.

10.6084/m9.figshare.16458840 Supplemental Video 6: https://doi.org/10.6084/m9.figshare.16458840.

10.6084/m9.figshare.16458849 Supplemental Video 7: https://doi.org/10.6084/m9.figshare.16458849.

## GRANTS

This project was partly supported by a BBSRC Grant No. BB/T003146.

## DISCLOSURES

No conflicts of interest, financial or otherwise, are declared by the authors.

## AUTHOR CONTRIBUTIONS

W.-C.L. conceived and designed research; V.S., H.M.L., A.L.S., and W.-C.L. performed experiments; V.S., H.M.L., and W.C.L. analyzed data; V.S. and W.-C.L. interpreted results of experiments; V.S. and W.-C.L. prepared figures; V.S. and W.-C.L. drafted manuscript; V.S., H.M.L., A.L.S., and W.-C.L. edited and revised manuscript; V.S., H.M.L., A.L.S., and W.-C.L. approved final version of manuscript.

## References

[1] Dijkgraaf S. The functioning and significance of the lateral-line organs. Biol Rev Camb Philos Soc 38: 51–105, 1963. doi:10.1111/j.1469-185x.1963.tb00654.x. 14027866

[2] Coombs S, Zaidi ZH. The Mechanosensory Lateral Line: Neurobiology and Evolution. New York, NY: Springer, 2012. doi:10.1007/978-1-4612-3560-6.

[3] Bleckmann H, Zelick R. Lateral line system of fish. Integr Zool 4: 13–25, 2009. doi:10.1111/j.1749-4877.2008.00131.x. 21392273

[4] Webb JF. Morphological diversity, development, and evolution of the mechanosensory lateral line system. In: The Lateral Line System, edited by Coombs S, Bleckmann H, Fay RR, Popper AN. New York, NY: Springer, 2014, vol. 48, p. 17–72.

[5] Suli A, Watson GM, Rubel EW, Raible DW. Rheotaxis in larval zebrafish is mediated by lateral line mechanosensory hair cells. PLoS One 7: e29727, 2012. doi:10.1371/journal.pone.0029727. 22359538 PMC3281009

[6] Oteiza P, Odstrcil I, Lauder G, Portugues R, Engert F. A novel mechanism for mechanosensory-based rheotaxis in larval zebrafish. Nature 547: 445–448, 2017. doi:10.1038/nature23014. 28700578 PMC5873946

[7] Montgomery JC, Baker CF, Carton AG. The lateral line can mediate rheotaxis in fish. Nature (London) 389: 960–963, 1997. doi:10.1038/40135.

[8] Simmons AM, Costa LM, Gerstein HB. Lateral line-mediated rheotactic behavior in tadpoles of the African clawed frog (*Xenopus laevis*). J Comp Physiol A Neuroethol Sens Neural Behav Physiol 190: 747–758, 2004. doi:10.1007/s00359-004-0534-3. 15300386 PMC1193646

[9] Roberts A, Feetham B, Pajak M, Teare T. Responses of hatchling *Xenopus* tadpoles to water currents: first function of lateral line receptors without cupulae. J Exp Biol 212: 914–921, 2009. doi:10.1242/jeb.027250. 19282488

[10] Stewart WJ, Cardenas GS, McHenry MJ. Zebrafish larvae evade predators by sensing water flow. J Exp Biol 216: 388–398, 2013. doi:10.1242/jeb.072751. 23325859

[11] McHenry MJ, Feitl KE, Strother JA, Van Trump WJ. Larval zebrafish rapidly sense the water flow of a predator's strike. Biol Lett 5: 477–479, 2009. doi:10.1098/rsbl.2009.0048. 19324627 PMC2781903

[12] Olszewski J, Haehnel M, Taguchi M, Liao JC. Zebrafish larvae exhibit rheotaxis and can escape a continuous suction source using their lateral line. PLoS One 7: e36661, 2012. doi:10.1371/journal.pone.0036661. 22570735 PMC3343021

[13] Claas B, Munz H. Analysis of surface wave direction by the lateral line system of *Xenopus*: source localization before and after inactivation of different parts of the lateral line. J Comp Physiol A 178: 253–268, 1996. doi:10.1007/BF00188167. 8592306

[14] Pohlmann K, Grasso FW, Breithaupt T. Tracking wakes: the nocturnal predatory strategy of piscivorous catfish. Proc Natl Acad Sci USA 98: 7371–7374, 2001. doi:10.1073/pnas.121026298. 11390962 PMC34675

[15] Pohlmann K, Atema J, Breithaupt T. The importance of the lateral line in nocturnal predation of piscivorous catfish. J Exp Biol 207: 2971–2978, 2004. doi:10.1242/jeb.01129. 15277552

[16] Denton EJ, Gray J. Mechanical factors in the excitation of clupeid lateral lines. Proc R Soc Lond B Biol Sci 218: 1–26, 1983. doi:10.1098/rspb.1983.0023. 6135206

[17] Gray J. Interaction of sound pressure and particle acceleration in the excitation of the lateral-line neuromasts of sprats. Proc R Soc Lond B Biol Sci 220: 299–325, 1984. doi:10.1098/rspb.1984.0002.

[18] Partridge BL, Pitcher TJ. The sensory basis of fish schools: relative roles of lateral line and vision. J Comp Physiol 135: 315–325, 1980. doi:10.1007/BF00657647.

[19] Katz LC, Potel MJ, Wassersug RJ. Structure and mechanisms of schooling in tadpoles of the clawed frog, *Xenopus laevis*. Anim Behav 29: 20–33, 1981. doi:10.1016/S0003-3472(81)80148-0.

[20] Flock Å. The lateral line organ mechanoreceptors. In: Fish Physiology, edited by Hoar WS, Randall DJ. New York, NY: Academic Press, 1971, p. 241–263.

[21] Lannoo MJ. The evolution of the amphibian lateral line system and its bearing on amphibian phylogeny. J Zool Syst Evol Res 26: 128–134, 2009. doi:10.1111/j.1439-0469.1988.tb00304.x.

[22] Flock Å. Transducing mechanisms in the lateral line canal organ receptors. Cold Spring Harb Symp Quant Biol 30: 133–145, 1965. doi:10.1101/sqb.1965.030.01.016. 5219466

[23] Strelioff D, Honrubia V. Neural transduction in *Xenopus laevis* lateral line system. J Neurophysiol 41: 432–444, 1978. doi:10.1152/jn.1978.41.2.432. 650276

[24] Kishida R, Goris RC, Nishizawa H, Koyama H, Kadota T, Amemiya F. Primary neurons of the lateral line nerves and their central projections in hagfishes. J Comp Neurol 264: 303–310, 1987. doi:10.1002/cne.902640303. 3680634

[25] Koester DM. Central projections of the octavolateralis nerves of the clearnose skate, *Raja eglanteria*. J Comp Neurol 221: 199–215, 1983. doi:10.1002/cne.902210208. 6655082

[26] Koyama H, Kishida R, Goris RC, Kusunoki T. Organization of the primary projections of the lateral line nerves in the lamprey *Lampetra japonica*. J Comp Neurol 295: 277–289, 1990. doi:10.1002/cne.902950210. 2358517

[27] McCormick CA. Central lateral line mechanosensory pathways in bony fish. In: The Mechanosensory Lateral Line, edited by Coombs S, Görner P, Münz H. New York, NY: Springer, 1989, p. 341–364. doi:10.1007/978-1-4612-3560-6_17.

[28] Puzdrowski RL. Peripheral distribution and central projections of the lateral-line nerves in goldfish, *Carasius auratus* (Part 1 of 2). Brain Behav Evol 34: 110–120, 1989. doi:10.1159/000116496. 2819411

[29] Puzdrowski RL, Leonard RB. The octavolateral systems in the stingray, *Dasyatis sabina*. I. Primary projections of the octaval and lateral line nerves. J Comp Neurol 332: 21–37, 1993. doi:10.1002/cne.903320103. 8514920

[30] Ronan M, Northcutt RG. Primary projections of the lateral line nerves in adult lampreys. Brain Behav Evol 30: 62–81, 1987. doi:10.1159/000118638. 3620897

[31] Fritzsch B. Diversity and regression in the amphibian lateral line and electrosensory system. In: The Mechanosensory Lateral Line, edited by Coombs S, Görner P, Münz H. New York, NY: Springer, 1989, p. 99–114. doi:10.1007/978-1-4612-3560-6_5.

[32] Shelton PM. The lateral line system at metamorphosis in *Xenopus laevis* (Daudin). J Embryol Exp Morphol 24: 511–524, 1970. 5493274

[33] Quinzio S, Fabrezi M. The lateral line system in anuran tadpoles: neuromast morphology, arrangement, and innervation. Anat Rec (Hoboken) 297: 1508–1522, 2014. doi:10.1002/ar.22952. 24863412

[34] Schlosser G. Development and evolution of lateral line placodes in amphibians. II. Evolutionary diversification. Zoology (Jena) 105: 177–193, 2002. doi:10.1078/0944-2006-00062. 16351867

[35] Schlosser G. Development and evolution of lateral line placodes in amphibians. I. Development. Zoology (Jena) 105: 119–146, 2002. doi:10.1078/0944-2006-00058. 16351862

[36] Russell IJ. Amphibian lateral line receptors. In: Frog Neurobiology: A Handbook, edited by Llinás R, Precht W. Berlin, Heidelberg: Springer, 1976, p. 513–550.

[37] Roberts A, Li WC, Soffe SR. How neurons generate behavior in a hatchling amphibian tadpole: an outline. Front Behav Neurosci 4: 16, 2010. doi:10.3389/fnbeh.2010.00016. 20631854 PMC2903309

[38] Roberts A, Li W-C, Soffe SR. A functional scaffold of CNS neurons for the vertebrates: the developing *Xenopus laevis* spinal cord. Dev Neurobiol 72: 575–584, 2012. doi:10.1002/dneu.20889. 21485014

[39] Kiehn O. Decoding the organization of spinal circuits that control locomotion. Nat Rev Neurosci 17: 224–238, 2016. doi:10.1038/nrn.2016.9. 26935168 PMC4844028

[40] Grillner S, El Manira A. Current principles of motor control, with special reference to vertebrate locomotion. Physiol Rev 100: 271–320, 2020. doi:10.1152/physrev.00015.2019. 31512990

[41] Koutsikou S, Merrison-Hort R, Buhl E, Ferrario A, Li WC, Borisyuk R, Soffe SR, Roberts A. A simple decision to move in response to touch reveals basic sensory memory and mechanisms for variable response times. J Physiol 596: 6219–6233, 2018. doi:10.1113/JP276356. 30074236 PMC6292811

[42] Roberts A, Borisyuk R, Buhl E, Ferrario A, Koutsikou S, Li WC, Soffe SR. The decision to move: response times, neuronal circuits and sensory memory in a simple vertebrate. Proc Biol Sci 286: 20190297, 2019. doi:10.1098/rspb.2019.0297. 30900536 PMC6452071

[43] Clarke JDW, Hayes BP, Hunt SP, Roberts A. Sensory physiology, anatomy and immunohistochemistry of Rohon-Beard neurones in embryos of *Xenopus laevis*. J Physiol 348: 511–525, 1984. doi:10.1113/jphysiol.1984.sp015122. 6201612 PMC1199414

[44] Pisano GC, Mason SM, Dhliwayo N, Intine RV, Sarras MP Jr. An assay for lateral line regeneration in adult zebrafish. J Vis Exp (86): e51343, 2014. doi:10.3791/51343. PMC4401111 24747778

[45] Schindelin J, Arganda-Carreras I, Frise E, Kaynig V, Longair M, Pietzsch T, Preibisch S, Rueden C, Saalfeld S, Schmid B, Tinevez JY, White DJ, Hartenstein V, Eliceiri K, Tomancak P, Cardona A. Fiji: an open-source platform for biological-image analysis. Nat Methods 9: 676–682, 2012. doi:10.1038/nmeth.2019. 22743772 PMC3855844

[46] Nieuwkoop PD, Faber J. Normal Tables of Xenopus laevis (Daudin): A Systematical and Chronologica Survey of the Development from the Fertilized Egg Till the End of Metamorphosis. Amsterdam: North Holland Pub Co., 1956.

[47] Li WC, Zhu XY, Ritson E. Mechanosensory stimulation evokes acute concussion-like behavior by activating GIRKs coupled to muscarinic receptors in a simple vertebrate. eNeuro 4: ENEURO.0073-17.2017, 2017. doi:10.1523/ENEURO.0073-17.2017. 28462392 PMC5409982

[48] Kahn JA, Roberts A, Kashin SM. The neuromuscular basis of swimming movements in embryos of the amphibian. J Exp Biol 99: 175–184, 1982. 7130896 10.1242/jeb.99.1.175

[49] Ritson EJ, Li W-C. The neuronal mechanisms underlying locomotion termination. Curr Opin Physiol 8: 109–115, 2019. doi:10.1016/j.cophys.2019.01.009.

[50] Li WC, Merrison-Hort R, Zhang HY, Borisyuk R. The generation of antiphase oscillations and synchrony by a rebound-based vertebrate central pattern generator. J Neurosci 34: 6065–6077, 2014. doi:10.1523/JNEUROSCI.4198-13.2014. 24760866 PMC3996224

[51] Naumann B, Olsson L. Three-dimensional reconstruction of the cranial and anterior spinal nerves in early tadpoles of *Xenopus laevis* (Pipidae, Anura). J Comp Neurol 526: 836–857, 2018. doi:10.1002/cne.24370. 29218708

[52] Roberts A. Pineal eye and behaviour in *Xenopus* tadpoles. Nature 273: 774–775, 1978. doi:10.1038/273774a0. 661985

[53] Jamieson D, Roberts A. Responses of young *Xenopus laevis* tadpoles to light dimming: possible roles for the pineal eye. J Exp Biol 203: 1857–1867, 2000. 10821743 10.1242/jeb.203.12.1857

[54] Van Trump WJ, Coombs S, Duncan K, McHenry MJ. Gentamicin is ototoxic to all hair cells in the fish lateral line system. Hear Res 261: 42–50, 2010. doi:10.1016/j.heares.2010.01.001. 20060460

[55] Coffin AB, Ramcharitar J. Chemical ototoxicity of the fish inner ear and lateral line. Adv Exp Med Biol 877: 419–437, 2016. doi:10.1007/978-3-319-21059-9_18. 26515324

[56] Chagnaud BP, Banchi R, Simmers J, Straka H. Spinal corollary discharge modulates motion sensing during vertebrate locomotion. Nat Commun 6: 7982, 2015. doi:10.1038/ncomms8982. 26337184 PMC4569702

[57] Roberts BL, Russell IJ. The activity of lateral-line efferent neurones in stationary and swimming dogfish. J Exp Biol 57: 435–448, 1972. 4634495 10.1242/jeb.57.2.435

[58] Soffe SR, Roberts A, Li W-C. Defining the excitatory neurons that drive the locomotor rhythm in a simple vertebrate: insights into the origin of reticulospinal control. J Physiol 587: 4829–4844, 2009. doi:10.1113/jphysiol.2009.175208. 19703959 PMC2770150

[59] Tunstall MJ, Roberts A. Longitudinal coordination of motor output during swimming in *Xenopus* embryos. Proc Biol Sci 244: 27–32, 1991. doi:10.1098/rspb.1991.0046. 1677193

[60] Carreno CA, Nishikawa KC. Aquatic feeding in pipid frogs: the use of suction for prey capture. J Exp Biol 213: 2001–2008, 2010. doi:10.1242/jeb.043380. 20511513 PMC2878287

[61] Fetcho J, Faber D. Identification of motoneurons and interneurons in the spinal network for escapes initiated by the Mauthner cell in goldfish. J Neurosci 8: 4192–4213, 1988. doi:10.1523/JNEUROSCI.08-11-04192.1988. 3183720 PMC6569477

[62] Faber DS, Fetcho JR, Korn H. Neuronal networks underlying the escape response in goldfish. General implications for motor control. Ann NY Acad Sci 563: 11–33, 1989. doi:10.1111/j.1749-6632.1989.tb42187.x. 2672948

[63] Fetcho JR. Spinal network of the Mauthner cell. Brain Behav Evol 37: 298–316, 1991. doi:10.1159/000114367. 1933252

[64] Kohashi T, Nakata N, Oda Y. Effective sensory modality activating an escape triggering neuron switches during early development in zebrafish. J Neurosci 32: 5810–5820, 2012. doi:10.1523/JNEUROSCI.6169-11.2012. 22539843 PMC6703610

[65] Mirjany M, Faber DS. Characteristics of the anterior lateral line nerve input to the Mauthner cell. J Exp Biol 214: 3368–3377, 2011. doi:10.1242/jeb.056226. 21957100

[66] van Mier P, ten Donkelaar HJ. Early development of descending pathways from the brain stem to the spinal cord in *Xenopus laevis*. Anat Embryol (Berl) 170: 295–306, 1984. doi:10.1007/BF00318733. 6335361

[67] Sillar KT, Robertson RM. Thermal activation of escape swimming in post-hatching *Xenopus laevis* frog larvae. J Exp Biol 212: 2356–2364, 2009. doi:10.1242/jeb.029892. 19617428 PMC2712414

[68] Quick QA, Serrano EE. Inner ear formation during the early larval development of *Xenopus laevis*. Dev Dyn 234: 791–801, 2005. doi:10.1002/dvdy.20610. 16217737 PMC2829094

[69] Will U. Amphibian Mauthner cells. Brain Behav Evol 37: 317–332, 1991. doi:10.1159/000114368. 1657273

[70] Eaton RC, Hackett JT. The role of the Mauthner cell in fast-starts involving escape in teleost fishes. In: Neural Mechanisms of Startle Behavior, edited by Eaton RC. Boston, MA: Springer, 1984, p. 213–266. doi:10.1007/978-1-4899-2286-1_8.

[71] Bin JM, Lyons DA. Imaging myelination in vivo using transparent animal models. Brain Plast 2: 3–29, 2016. doi:10.3233/BPL-160029. 29765846 PMC5928531

[72] Chase MH, Wills N. Brain stem control of masseteric reflex activity during sleep and wakefulness: medulla. Exp Neurol 64: 118–131, 1979. doi:10.1016/0014-4886(79)90009-8. 428491

[73] Pearson KG, Collins DF. Reversal of the influence of group Ib afferents from plantaris on activity in medial gastrocnemius muscle during locomotor activity. J Neurophysiol 70: 1009–1017, 1993. doi:10.1152/jn.1993.70.3.1009. 8229157

[74] Voigt R, Carton AG, Montgomery JC. Responses of anterior lateral line afferent neurones to water flow. J Exp Biol 203: 2495–2502, 2000. doi:10.1242/jeb.203.16.2495. 10903164

[75] Mensinger AF, Van Wert JC, Rogers LS. Lateral line sensitivity in free-swimming toadfish *Opsanus tau*. J Exp Biol 222: jeb190587, 2019. doi:10.1242/jeb.190587. 30446535

[76] Pichler P, Lagnado L. The transfer characteristics of hair cells encoding mechanical stimuli in the lateral line of zebrafish. J Neurosci 39: 112–124, 2019. doi:10.1523/JNEUROSCI.1472-18.2018. 30413644 PMC6325263

[77] Haehnel-Taguchi M, Akanyeti O, Liao JC. Afferent and motoneuron activity in response to single neuromast stimulation in the posterior lateral line of larval zebrafish. J Neurophysiol 112: 1329–1339, 2014. doi:10.1152/jn.00274.2014. 24966296 PMC4137249

[78] Brenner N, Bialek W, de Ruyter van Steveninck R. Adaptive rescaling maximizes information transmission. Neuron 26: 695–702, 2000. doi:10.1016/s0896-6273(00)81205-2. 10896164

[79] Wark B, Lundstrom BN, Fairhall A. Sensory adaptation. Curr Opin Neurobiol 17: 423–429, 2007. doi:10.1016/j.conb.2007.07.001. 17714934 PMC2084204

[80] Schnee ME, Santos-Sacchi J, Castellano-Muñoz M, Kong JH, Ricci AJ. Calcium-dependent synaptic vesicle trafficking underlies indefatigable release at the hair cell afferent fiber synapse. Neuron 70: 326–338, 2011. doi:10.1016/j.neuron.2011.01.031. 21521617 PMC3254016

[81] Goutman JD. Mechanisms of synaptic depression at the hair cell ribbon synapse that support auditory nerve function. Proc Natl Acad Sci USA 114: 9719–9724, 2017. doi:10.1073/pnas.1706160114. 28827351 PMC5594669

[82] Northcutt RG. The phylogenetic distribution and innervation of craniate mechanoreceptive lateral lines. In: The Mechanosensory Lateral Line, edited by Coombs S, Görner P, Münz H. New York, NY: Springer, 1989, p. 17–78. doi:10.1007/978-1-4612-3560-6_3.

[83] Boord RL, Montgomery JC. Central mechanosensory lateral line centers and pathways among the elasmobranchs. In: The Mechanosensory Lateral Line, edited by Coombs S, Görner P, Münz H. New York, NY: Springer, 1989, p. 323–339. doi:10.1007/978-1-4612-3560-6_16.

[84] Boord RL, Northcutt RG. Ascending lateral line pathways to the midbrain of the clearnose skate, *Raja eglanteria*. J Comp Neurol 207: 274–282, 1982. doi:10.1002/cne.902070307. 7107987

[85] McCormick CA, Hernandez DV. Connections of octaval and lateral line nuclei of the medulla in the goldfish, including the cytoarchitecture of the secondary octaval population in goldfish and catfish (Part 1 of 2). Brain Behav Evol 47: 113–125, 1996. doi:10.1159/000113232. 8680846

[86] Wullimann MF, Grothe B. The central nervous organization of the lateral line system. In: The Lateral Line System, edited by Coombs S, Bleckmann H, Fay RR, Popper AN. New York, NY: Springer, 2014, p. 195–251. doi:10.1007/2506_2013_18.

[87] Will U. Central mechanosensory lateral line system in amphibians. In: The Mechanosensory Lateral Line, edited by Coombs S, Görner P, Münz H. New York, NY: Springer, 1989, p. 365–386. doi:10.1007/978-1-4612-3560-6_18.

[88] Fritzsch B, Nikundiwe AM, Will U. Projection patterns of lateral-line afferents in anurans: a comparative HRP study. J Comp Neurol 229: 451–469, 1984. doi:10.1002/cne.902290312. 6209307

[89] Bricaud O, Chaar V, Dambly-Chaudiere C, Ghysen A. Early efferent innervation of the zebrafish lateral line. J Comp Neurol 434: 253–261, 2001. doi:10.1002/cne.1175. 11331527

[90] Metcalfe WK, Kimmel CB, Schabtach E. Anatomy of the posterior lateral line system in young larvae of the zebrafish. J Comp Neurol 233: 377–389, 1985. doi:10.1002/cne.902330307. 3980776

[91] Tricas TC, Highstein SM. Visually mediated inhibition of lateral line primary afferent activity by the octavolateralis efferent system during predation in the free-swimming toadfish, *Opsanus tau*. Exp Brain Res 83: 233–236, 1990. doi:10.1007/BF00232215. 2073946

[92] Montgomery JC, Bodznick D. An adaptive filter that cancels self-induced noise in the electrosensory and lateral line mechanosensory systems of fish. Neurosci Lett 174: 145–148, 1994. doi:10.1016/0304-3940(94)90007-8. 7970170

[93] Lunsford ET, Skandalis DA, Liao JC. Efferent modulation of spontaneous lateral line activity during and after zebrafish motor commands. J Neurophysiol 122: 2438–2448, 2019. doi:10.1152/jn.00594.2019. 31642405 PMC6966311

[94] Hellmann B, Fritzsch B. Neuroanatomical and histochemical evidence for the presence of common lateral line and inner ear efferents and of efferents to the basilar papilla in a frog, *Xenopus laevis*. Brain Behav Evol 47: 185–194, 1996. doi:10.1159/000113238. 9156781

[95] Roberts BL, Meredith GE. The efferent system. In: The Mechanosensory Lateral Line, edited by Coombs S, Görner P, Münz H. New York, NY: Springer, 1989, p. 445–459.

[96] Chagnaud BP, Coombs S. Information encoding and processing by the peripheral lateral line system. In: The Lateral Line System, edited by Coombs S, Bleckmann H, Fay RR, Popper AN. New York, NY: Springer, 2014, p. 151–194.

[97] Lowe DA, Russell IJ. The central projections of lateral line and cutaneous sensory fibers in *Xenopus laevis*. Proc R Soc Lond B 216: 279–297, 1982. doi:10.1098/rspb.1982.0075. 6129631

[98] Will U. Efferent neurons of the lateral line system and the VIIIth cranial nerve in the brainstem of anurans. Cell Tissue Res 225: 673–685, 1982. doi:10.1007/BF00214812. 6181890

[99] Flock A, Lam DM. Neurotransmitter synthesis in inner ear and lateral line sense organs. Nature 249: 142–144, 1974. doi:10.1038/249142a0. 4151611

[100] Sillar KT, Combes D, Simmers J. Neuromodulation in developing motor microcircuits. Curr Opin Neurobiol 29: 73–81, 2014. doi:10.1016/j.conb.2014.05.009. 24967995

